# Antibacterial Efficacy of Graphene Nanoparticles against *Enterococcus faecalis* : *In Vitro* Study

**DOI:** 10.1055/s-0044-1786863

**Published:** 2024-07-08

**Authors:** Omer Sheriff Sultan, Preena Sidhu, Kiran Rehman, Thiagrajan Madheswaran, Amalraj Fabian Davamani

**Affiliations:** 1 A.T. Still University - Missouri School of Dentistry and Oral Health (ATSU-MOSDOH), Kirksville, Missouri, United States; 2 Division of Restorative Dentistry, School of Dentistry, International Medical University, Kuala Lumpur, Malaysia; 3 Department of Pharmaceutical Technology, School of Pharmacy, International Medical University, Kuala Lumpur, Malaysia; 4 School of Health Sciences, International Medical University, Kuala Lumpur, Malaysia

**Keywords:** antimicrobial, chlorhexidine, calcium hydroxide, *Enterococcus faecalis*, nanographene, root canal therapy

## Abstract

**Objective(s)** This study compared the antimicrobial efficacy of nanographene (NG) particles with chlorhexidine (CHX) and calcium hydroxide (Ca(OH) _2_ ) against *Enterococcus faecalis* .

**Materials and Methods** Forty extracted human mandibular premolar teeth were cleaned using a scaler, and the middle-third of the root (6 mm) was decoronated using a rotary diamond disk. The inner diameter of the teeth was made consistent using Gates Glidden Drills #3, treated with ethylene diamine tetra-acetic acid and sodium hypochlorite before sterilization. The samples were then contaminated with *E. faecalis* grown in Tryptic soy broth for 21 days. Tooth samples were then randomly divided into four groups: Group I (Control), untreated saline; Group II, Ca(OH) _2_ ; Group III, CHX; and Group IV, NG. The assessment of bacterial growth was carried out by harvesting dentin chips at the end of 1, 3, and 7 days. The colonies were physically counted and tabulated after 24 hours from seeding. Statistical analysis of the collected data was performed with analysis of variance and Tukey's post hoc test using SPSS Version 20.0.

**Results** The contaminated dentine blocks irrigated with NG (0.5 µg) and CHX (0 ± 0; *p* < 0.001) had no growth of *E. faecalis* colonies compared to blocks of Ca(OH) _2_ (10 ± 21) and saline (927 ± 455). All concentrations of NG (0.5 and 1.0 µg) showed effectiveness higher ( *p* < 0.001) than 2% CHX when measured by the zone of inhibition against *E. faecalis.*

**Conclusion** It may be concluded that NG is effective against growth of *E. faecalis* and may be used as a promising antimicrobial agent during root canal treatment. However, further studies should be done to investigate the effect of NG against other dental pathogens.

## Introduction

Pulpal and periapical pathology are primarily caused by microbial insults and the presence of microorganisms in dentinal tubules leading to the recurrence of apical periodontitis. Hence, the objective of nonsurgical root canal treatment is to eliminate microorganisms from the root canal system to prevent failure. The combined use of instrumentation and irrigants helps to reduce the microorganisms. After instrumentation, 35% or more of the canal's surface area remain unchanged, which could be the limitation of the instrumentation technique. 1 The mechanical cleaning with irrigation using antimicrobials could significantly decrease the bacterial load in the root canal system, 2 but residual bacteria could still flourish and multiply in between appointments if no antimicrobial agent was applied in the root canal system. Hence, intracanal medicament plays a key role in root canal treatment. 3

Failures of nonsurgical root canal therapy are frequently associated with the presence of *Enterococcus faecalis* in root canal systems. 4 5 Close to the pulp and outer borders of the tooth, the diameter of dentinal tubules are 2.5 µm and 0.95 µm, respectively. However, the mean diameter of *E. faecalis* is between 0.8 and 1 µm, which allows the organism to enter and survive within the dentinal tubules and form biofilm on walls of root canals. 6 7 8 9 Maximum penetration of *E. faecalis* was found to be 1,483.33 µm. Hence, the penetrating ability of the traditional antimicrobial agents needs to be higher to reduce the remaining bacteria inside the dentinal tubules. 10

Calcium hydroxide (Ca(OH) _2_ ) and chlorhexidine (CHX) gluconate are commonly used as intracanal medicaments in endodontic therapy. The mode of action of Ca(OH) _2_ allows it to be a physical barrier, disturbing remaining nutrient resources for bacteria, 8 and most importantly a high pH of 12.5 causes lysis of bacterial cell membrane and proteins. 11 CHX's mode of action is through penetration of negatively charged bacteria cell wall by the positively charged CHX molecules that are toxic to bacteria. 11 12

Graphene is an atomically thin two-dimensional layer. It has a large specific surface area and is highly suited to be a promising drug carrier. 13 14 15 It also has unique chemical and physical properties that make it possible to react with molecules and modify the surfaces leading to important molecules like graphene oxide (GO) and carboxylated graphene. 16 17 Graphene, an allotropic type of carbon, was first successfully prepared by Novoselov et al in 2004. 18 Graphene is constituted of single-atom-thick carbon nanosheets with a honeycomb structure.

Singular graphene units are called nanographenes (NGs); NGs are graphene fragments with a diameter of less than 100 nm, while graphene should exceed 100 nm in both directions. The production of NG is quite complicated: it is done by a process called dehydrogenation. Dehydrogenation is achieved by selectively removing hydrogen atoms from organic molecules consisting of carbon and hydrogen.

Potential applications of graphene and its derivatives in biomedical fields such drug delivery, tissue engineering, and biosensors have been investigated. Applications of nanotechnology in endodontics include the incorporation of bio-ceramic nanoparticles such as bioglass, zirconia, and glass ceramics in endodontic sealers. It has been found that the use of nanoparticles enhances the adaptation of the adhesive to nano-irregularities, in addition to its fast setting time in comparison to conventional sealers, its dimensional stability, insolubility in tissue fluid, chemical bond to tooth tissue, and osseo conductivity. 19 The use of nanoparticles in dentistry is gaining popularity for various applications. 20 21 Numerous studies have investigated the effect of NG in dentistry including endodontics. 22 23

The effectiveness of NG oxide as intracanal medications has been evaluated in earlier research. 21 Nevertheless, no research has been done on the use of NG as an intracanal irrigant.

The objective of this study was to compare the antimicrobial efficacy of NG particles compared to CHX and Ca(OH) _2_ against *E. faecalis* in contaminated extracted teeth using colony-forming unit (CFU).

## Materials and Methods

A randomized controlled *in vitro* study was designed. The study protocol was approved by the institutional research ethical committee (IMU-JC NO 361/2016). The minimum inhibitory concentration (MIC) of NG against *E. faecalis* was determined using serial dilutions of 0.1%, 2% CHX, and 3% sodium hypochlorite (NaOCl). Cultures of *E. faecalis* was prepared to obtain the 0.5 McFarland turbidity. The MIC was defined as the lowest concentration of each irrigant in which no visible turbidity was noted.

NG particles powder (Sigma Aldrich Gold nano urchins) was mixed in saline to form a suspension.

Each treatment (untreated × 3; NG 1,000 µg/mL; NG 750 µg/mL; NG 500 µg/mL; NG 250 µg/mL; NG 200 µg/mL; NG 150 µg/mL; NG 100 µg/mL; NG 50 µg/mL) was then added accordingly to the corresponding wells.

### Sample Preparation for Tooth Model

Forty extracted human mandibular premolar teeth were used for this project. A pilot study was conducted in our experimental research to calculate the sample size accurately using G*power (Version 3.1)

Accordingly, a total of 40 extracted human mandibular premolars were used in this study that were further divided into 4 main groups consisting of 10 samples for each group, in accordance with the previously calculated sample size.

The external surfaces of the teeth were cleaned using an ultrasonic scaler. A rotary diamond disk was used to obtain the middle-third of the root (6 mm) by decoronating the teeth below the cementoenamel junction and the apical part of the root. The accuracy of the slices was checked with calipers. The inner diameter of the teeth was made consistent using #3 Gates Glidden (GG) (Mani Inc, Tochigi-ken, Japan). To remove debris, the teeth samples were placed in an ultrasonic bath containing 17% ethylene diamine tetra-acetic acid (PD Dental) for 5 minutes followed by 3% NaOCl (Coltene). Samples were placed in an ultrasonic distilled water bath for three cycles of 20 minutes. Lastly, the teeth samples were sterilized by autoclave (Infitek Class B benchtop sterilizer).

### Sample Contamination

*E. faecalis* (ATCC 29212) cells were grown in Tryptic soy broth (TSB) overnight. After incubation, the culture was diluted in 5 mL of TSB and its turbidity adjusted to 0.5 McFarland standards (1 × 10 ^6^ CFU/mL). Each dentine block was immersed in presterilized microcentrifuge tubes containing TSB. To contaminate the dentine blocks, 50 µL of the overnight *E. faecalis* culture was transferred into each of the microcentrifuge tubes. The purity of the culture was checked by subculturing 5 µL of the broth from the incubated dentine blocks on Tryptic soy agar (TSA) plates. Dentine blocks were contaminated with *E. faecalis* for 21 days, where media was replaced daily.

### Antibacterial Assessment

After incubation (21 days), the tooth samples were washed with 5 mL of sterile saline to remove excess culture on the surface of the specimens. The teeth samples were randomly divided into 4 groups with 10 specimens each: Group I, untreated saline (control); Group II, Ca(OH) _2_ ; Group III, CHX; and Group IV, NG.

Preparation of NG gel was done by mixing 1 mg of NG powder with 1 mL of hydroxypropyl methylcellulose in a magnetic stirrer. Each treatment group was introduced into the teeth samples and paraffin wax was used to seal both ends of the dentine blocks. Incubation was carried out at 37°C for 24 hours. At the end of days 1, 3, and 7, an assessment of bacterial growth was carried out by harvesting dentine chips using GG #4 (200µm) into Eppendorf tubes containing 0.5 mL saline. Homogenization of the suspension was done through vortexing for 10 seconds. After this process, 0.05 mL from each Eppendorf tube was introduced into separate Eppendorf tubes of 0.45 mL saline. This process continued up to three micro dilutions for all treatment groups. The Eppendorf tubes holding dilutions 1, 2, and 3 for all variables had 0.02 mL of their contents drawn out and seeded onto the TSA plate in a triplicate pattern. The plates were then left to dry and incubated at 37°C for 24 hours. The colonies were physically counted and tabulated after 24 hours from seeding.

### Statistical Analysis

The collected data were analyzed with SPSS Version 20.0 by analysis of variance and Tukey's post hoc test. A *p* -value of less than 0.05 was considered to be statistically significant.

## Results

**Fig. 1:**
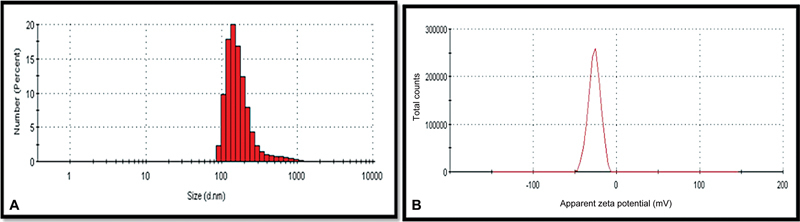
( **A** ) Size distribution intensity. ( **B** ) Zeta potential curve.

The average droplet and particle size distribution were analyzed using Zetasizer (Nano-ZS90, Malvern Instruments, Worcestershire, UK) ( Table 1 ). The surface charge of the formulations was also measured with the same instrument. As indicated previously, thermodynamic stability investigations on the NG were conducted. The graphene particle size analysis was done using Malvern Zetasizer ZS90, and the polydispersity index indicates the homogenous size distribution of the particles ( Fig. 1A ). If the value is around 0.5, it shows relative homogeneity. Zeta potential is the surface charge of a particle ( Fig. 1B ). If the observed surface charge is higher than –20, it indicates high stability of the particle.

**Table 1:** Physical characterization of nanographene

Nano formulation	Average particle size	Polydispersity index	Zeta potential
Nanographene	403.1 nm	0.541	–27.2 ± 0.7 mV

### Antibacterial Assessment

**Fig. 2:**
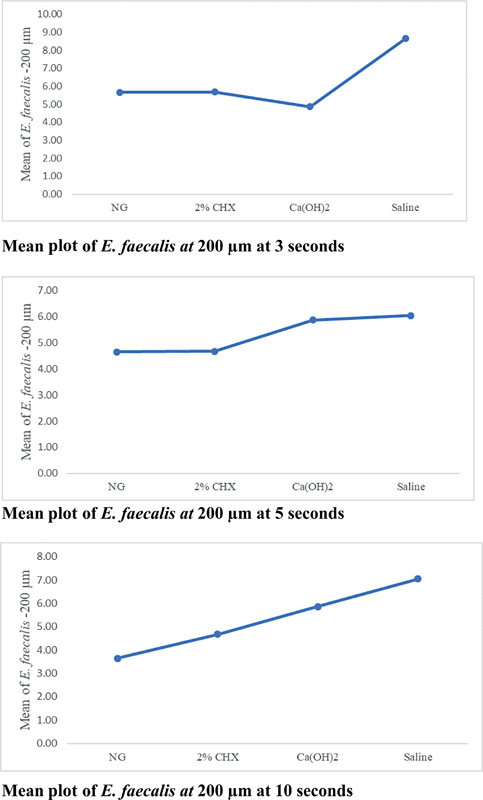
Mean plot of *Enterococcus faecalis at* 200 µm at varying time intervals. Ca(OH) _2,_ calcium hydroxide; CHX, chlorhexidine; NG, nanographene.

The contaminated dentine blocks irrigated with NG (0.5 µg) and 2% CHX (0 ± 0; *p* < 0.001) did not have any growth of *E. faecalis* colonies compared to blocks of Ca(OH) _2_ (10 ± 21) and saline (927 ± 455). All concentrations of NG (0.5 and 1.0 µg) showed effectiveness higher ( *p* < 0.001) than 2% CHX when measured by the zone of inhibition against *E. faecalis* ( Fig. 2 ).

Real-time polymerase chain reaction (PCR) was performed using a thermal cycler (7900 HT RT-PCR, Applied Biosystem, UK) 22 with SYBR™ Green fluorophore. The reaction mix was prepared to a final volume of 20 μL and loaded in an optical 96 well plate, which was then covered with an optical adhesive sheet. *E. faecalis* was identified using PCR amplification of 16S rRNA gene sequences. The oligonucleotide species-specific primers for *E. faecalis* were 5¢GTT TAT GCC GCA TGG CAT AAG AG3¢ (forward primer, located at base position 165 to 187 of the *E. faecalis* 16S rDNA) and 5¢CCG TCA GGG GAC GTT CAG¢3 (reverse primer, located at base position 457 to 474 of the *E. faecalis* 16S rDNA).

### Biocompatibility Assay

**Fig. 3:**
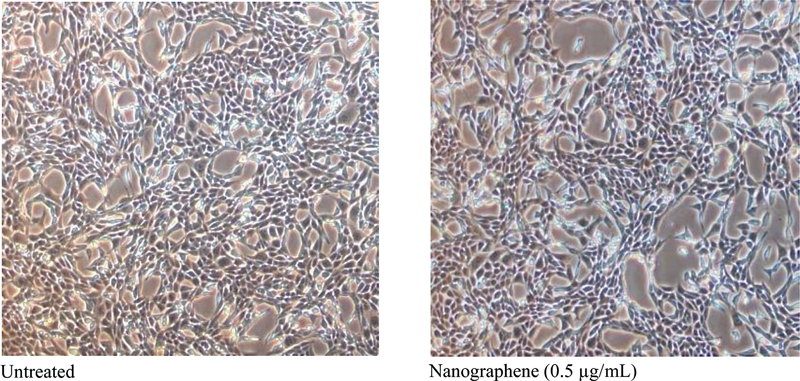
Cells in the presence and absence of nanographene in an NIH-3T3 (mouse fibroblast).

Figure 3 shows that the biocompatibility assay using epithelial and fibroblast cell lines was done using Trypan blue exclusion. Dilution of a cell sample in Trypan blue dye of an acid azo exclusion medium prepared by a 1:1 dilution of the cell suspension using a 0.4% Trypan blue solution was done. Nonviable cells will be blue while viable cells will be unstained. The total number of cells overlying 1 mm ^2^ was between 20 and 50 cells/square. The Trypan blue dye stains the dead cells blue that can be counted versus the live cells and the ratio can be used to see the percentage of dead cells (a small percentage of dead cells are likely; however, untreated sample and the treated sample should be compared to optimize the data).

## Discussion

Successful root canal therapy may be characterized as absence of clinical symptoms, regression of periapical lesion, tight seal canal and coronal spaces, and recovery of tooth function. 4 This can be achieved by root canal preparation, stringent chemo-mechanical debridement, and intracanal dressing placement, which aim to disrupt bacteria biofilm and their by-product along with neutralizing bacterial invasion within the complex root canal system. It is later followed by adequate sealing of canal spaces and restoration of root treated teeth to complete the treatment. 8 10 21 Different types of antimicrobials either single or in combination have been used in root canal disinfection. These antimicrobials act by promoting slow destruction of the biofilm structure by destroying persistent cell or quorum sensing signals, which then allows it to diffuse into the biofilm structure, signaling destruction of both the matrix and resident bacteria in the structure. 8 On the contrary, the increase in resistance against antimicrobials leads to persistence of certain species of bacteria namely *E. faecalis* , which have been commonly found to cause chronic inflammation in the root canal spaces and ultimately leading to endodontic failures. 7 11

This study investigated the antibacterial efficacy of NG against *E. faecalis* in comparison to Ca(OH) _2_ and CHX. Just like the cumulative nature of science, dentistry is ever evolving, into a new era of nanotechnology. New silver nanotechnology chemistry has also been proven to be effective against biofilms such as *Escherichia coli* , *Streptococcus pneumoniae* , *Staphylococcus aureus* , and *Aspergillus niger.* It is reported that silver has a high affinity against negatively charged side groups found distributed all over microbial cells and it acts by attacking multiple sites within the cell to inactivate its critical physiological functions. On top of that, it was also discovered that all it takes is as little as one part per billion of silver to be effective in preventing certain types of bacteria growth. 23

Our results show that contaminated dentin blocks irrigated with nanographene or CHX did not have any growth as compared to Ca(OH) _2_ and saline ( Fig. 2 ). The results reported are similar to studies that have evaluated the antibacterial efficacy of Ca(OH) _2_ using molecular method. Majority of the studies showed that although bacterial counts were significantly reduced, it was not completely eradicated. 24 It has been suggested that this could be due to the ability of microorganisms to stay hidden within the complexity of radicular canal spaces that have made the evasion of close contact with lethal hydroxyl ions possible. 25 26 On the contrary, in terms of clinical outcome, controversial results have been reported as there were no significant differences in healing modalities between single visits and multiple visits with or without interappointment Ca(OH) _2_ dressing. 27 28 In the present study, all concentrations of NG have shown effectiveness against *E. faecalis* and were found to be significantly higher than CHX. Similarly, in a study conducted by Wu et al, the antibacterial efficacy of GO was evaluated by fabricating a calcium phosphate cement-chitosan-GO scaffold and was tested against *E. faecalis* . 29 The results have shown that in the presence of graphene particles, it possesses high antibacterial activity against *E. faecalis* , hence rendering it a promising root canal sealer in endodontic treatment. Other than that, its versatility to undergo different surface modification and functionalization to reduce cytotoxicity along with its ability to be produced easily, and on large scale with low cost, makes GO a suitable antibacterial agent against multiresistant bacteria. 29

In our study, the antibacterial effect of CHX was found to be inferior to that of NG. This might be due to the resistance and poor penetration of CHX through the dense exopolysaccharides-matrix encased *E. faecalis* . 30

As opposed to NG, it has been found to exhibit strong antibacterial properties and this could be attributed to the ability of NG to impale the bacteria via physical and chemical mechanisms.

These mechanisms include the following:

1. Physically damage the bacterial membrane via direct contact.
2. Reactive oxygen species production causing disruption and deactivation of cell metabolism.
3. Electron transfer from bacterial membrane leading to compromised membrane integrity. 31

The application of nanoparticles into dentistry has been gaining popularity due to its excellent antibacterial properties. Its large surface to volume ratio, ultrasmall sizes, and excellent chemical and physical properties give graphene good bonding capabilities and surface chemistry as compared to conventional materials. 32 As a result, the interaction between the positively charged, increased surface area of nanoparticles and negatively charged bacterial cells leads to increased antibacterial activity.

To date, NaOCl remains the gold standard for root canal irrigation due to its potent antimicrobial and tissue dissolving properties; however, accidental apical extrusion may lead to inflammation and tissue cytotoxicity. 32

**Fig. 4:**
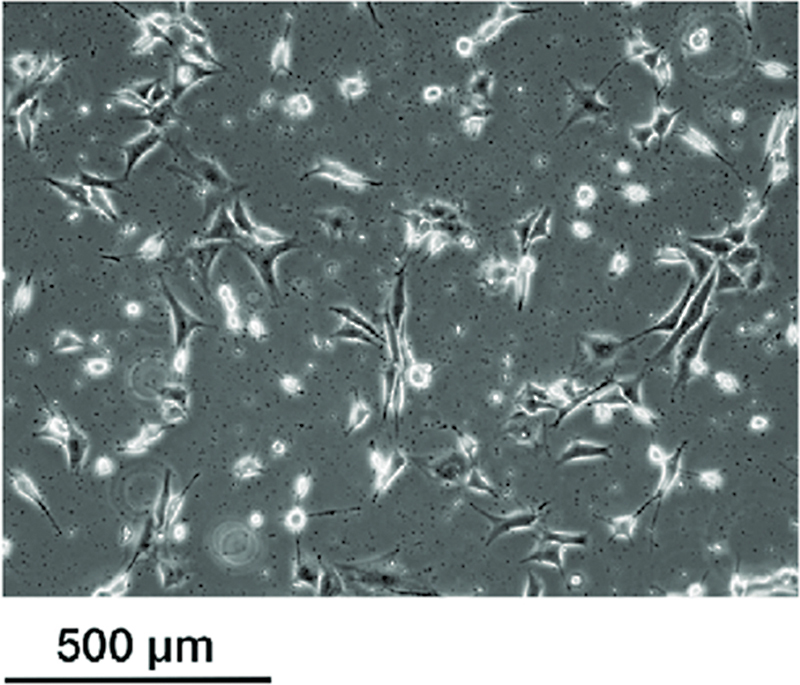
Dead cells appearing in the image shown treated with chlorhexidine.

Furthermore, it has been discovered that the antibacterial effect of graphene remains the same by incorporating it into silver nanoparticles while concurrently preserving its cytotoxic effect to surrounding tissues and bones. This can be supported by the findings from our study in which the epithelial and fibroblast cells treated with NG were found to be viable hence biocompatible to surrounding tissue structure ( Figs. 3 and 4 ). Multiple studies have also shown that monolayer graphene or GO films were found biocompatible to mouse fibroblasts (NIH-3T3), human osteoblasts-like cell line, and A549 cell line. 33 34 However, Zhang et al reported in a study that biocompatibility of NG platelets may be dose-dependent with 10 µg/mL ^−1^ as its critical concentration. 35 In addition to this, Chang et al 33 has reported that the cytotoxicity of graphene-based material may be affected by its shape and size in which the smaller the size of GO particles, the higher the cytotoxicity it expresses.

**Fig. 5:**
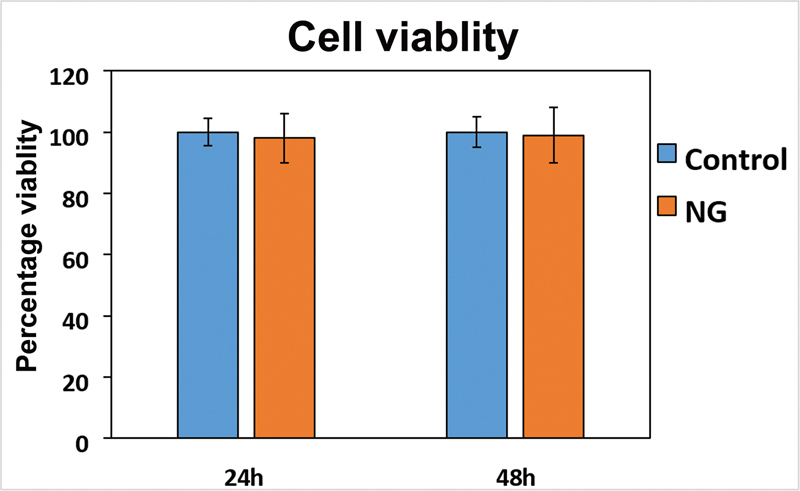
Cell viability is measured by live/dead staining of NIH-3T3 cells after incubation on each substrate for 24 and 48 hours. Live and dead cells were counted and the percentage of live cells was plotted. NG, nanographene.

From this we can deduce that even though smaller-sized graphene may enhance its ability to penetrate and impale bacteria, it may also be less biocompatible. Hence, more research should be conducted in formulating a size and dosage appropriate graphene-based material to be safely used in the human body. In our study, 0.5 µg/mL of NG has been shown to express potent antimicrobial activity against *E. faecalis* and was found to be biocompatible against mouse fibroblast cells (NIH-3T3) ( Fig. 5 ). From this, we can conclude that NG may be used as a promising antimicrobial agent in root canal treatment. Therefore, more research should be carried out to investigate the effect of NG against other dental pathogens such as *Streptococci* or *Porphyromonas* in *in vivo* or clinical settings.

## Conclusion

It may be concluded that NG is effective against growth of *E. faecalis* and may be used as a promising antimicrobial agent during root canal treatment. However, further studies should be done to investigate the effect of NG against other dental pathogens.

### Limitations and Future Recommendations

The limitations of the current study are that it assesses the antibacterial activity of NG in single-rooted premolars, and complex canal anatomies of multirooted teeth has not been evaluated. Future studies can assess the antibacterial effect of NG in multirooted teeth to assess the penetration into complex canal anatomy. Future studies can also investigate the mechanism of action and interaction with other intracanal medicaments and irrigants.
